# Knowledge, Attitudes, Perceptions, and Practices Related to Artificial Intelligence in Radiology Among Indian Radiologists and Residents: A Multicenter Nationwide Study

**DOI:** 10.7759/cureus.76667

**Published:** 2024-12-31

**Authors:** Swati Goyal, Pramod Sakhi, Sadhana Kalidindi, Deepal Nema, Abhijit P Pakhare

**Affiliations:** 1 Radiology, Gandhi Medical College, Bhopal, IND; 2 Radiodiagnosis, Sri Aurobindo Institute of Medical Sciences, Indore, IND; 3 AI: Public Health and Radiology, ARI Academy, Hyderabad, IND; 4 Community and Family Medicine, All India Institute of Medical Sciences, Bhopal, Bhopal, IND

**Keywords:** artificial intelligence, knowledge, physicians, radiology, residents

## Abstract

Background

Artificial Intelligence (AI) is revolutionizing medical science, with significant implications for radiology. Understanding the knowledge, attitudes, perspectives, and practices of medical professionals and residents related to AI's role in radiology is crucial for effective integration.

Methods

A cross-sectional survey was conducted among members of the Indian Radiology & Imaging Association (IRIA), targeting practicing radiologists and residents across academic and non-academic institutions. An anonymous, self-administered online questionnaire assessed AI awareness, usage, and perceptions, distributed via medical networks and social media. Descriptive statistics and chi-square tests were used to analyze the data, with statistical analysis performed using R version 4.2.2 (R Core Team (2021). R: A language and environment for statistical computing. R Foundation for Statistical Computing, Vienna, Austria. https://www.R-project.org/).

Results

The survey gathered responses from 404 participants nationwide. A significant portion (95.3%) demonstrated a keen interest in expanding their knowledge of AI and recommended implementing educational initiatives that increase exposure to AI. Considerable concern about losing their jobs to AI was observed only in 27.9% of respondents. More than two-thirds (86.6%) of the respondents opined that the AI curriculum should be taught during residency and 75.7% are interested in collaborating with software developers to learn and start AI at their workplace.

Conclusion

The survey highlights the growing importance of AI in radiology, underscoring the need for enhanced AI education and training in medical curricula.

## Introduction

In recent years, artificial intelligence (AI) has become a game-changer across various domains, particularly in the realm of medical sciences. Diagnostics, treatment planning, and patient care are being transformed by AI technologies driven by advancements in data analytics, machine learning, and deep learning [1]. Integrating AI into medical practice is more than just a technology enhancement; it signifies a fundamental change in approach, providing unparalleled accuracy and effectiveness [2].

Radiology, a field inherently reliant on image analysis, stands at the vanguard of this AI revolution. Deep learning-based AI algorithms have demonstrated exceptional competence in interpreting X-rays, CT scans, and MRIs, among other medical imaging modalities [3]. Improved diagnosis accuracy, faster reading times, and less room for human errors are all benefits of these technological advancements. Furthermore, AI's involvement in predictive analytics creates opportunities for personalized medicine, where treatment approaches are tailored to individual patient profiles [4].

Nevertheless, incorporating AI into radiology is full of stumbling blocks. Data privacy concerns, opaqueness/the lack of transparency in algorithms, and the necessity for reliable training datasets are relevant. Moreover, there is increasing apprehension regarding the influence of AI on jobs, specifically concerning the roles and responsibilities of radiologists [5,6].

Given these recent advancements, the objective of this survey is to assess the knowledge, awareness, and usage of AI technologies in radiology among radiologists and residents, comprehend their perceptions regarding the potential and prospective implications of AI in the field, and identify educational gaps to evaluate the necessity of incorporating AI-focused education and training into the medical curriculum, with a specific focus on radiology. The objective of the study is to assess the current knowledge, attitudes, perceptions, and practices of Indian radiologists and residents regarding AI in radiology. The broader implications of this study include informing policymakers, educators, and healthcare professionals to make well-informed decisions regarding AI use in radiology practices and education, aligning with recent research by Chen et al. [7].

## Materials and methods

Study design and setting

This study was conducted as a cross-sectional survey among members of the Indian Radiology & Imaging Association (IRIA). The survey targeted both practicing radiologists and radiology residents across various academic and non-academic institutions nationwide.

Participants

The study population included members of the Indian Radiology & Imaging Association (IRIA), with a diverse mix of radiology professionals from academic institutions, non-academic hospitals, and private practices across India. Academic participants were subcategorized into radiology residents (first-, second-, and third-year, and senior residents) and teaching faculty, including assistant professors, associate professors, professors, senior consultants, and others. Non-academic participants included practitioners from private diagnostic centers and hospitals.

Inclusion criteria encompassed active members of IRIA who were either teaching faculty or residents from academic institutes, as well as practitioners from non-academic settings specializing in diagnostic or interventional radiology. Conversely, individuals excluded from the study were non-members of IRIA and medical professionals not practicing radiology.

Data collection

An anonymous, self-administered online questionnaire was developed by investigators (Appendices). The questionnaire included information on demographic variables (age, gender), details of the radiology practice (such as type of institution, experience, designation, nature of services provided, etc), and a few multiple-choice and Likert-scale questions to assess various aspects of AI awareness, usage, and perception. The questionnaire was reviewed by a senior radiologist for relevance and accuracy before distribution.

The questionnaire was deployed on Google Forms. The link to the questionnaire was distributed online via emails registered in IRIA databases as well as through social media platforms (WhatsApp and Telegram groups). The survey was open for responses for four weeks. A reminder to participate was sent weekly to maximize participation.

The data collection was carried out between 01 June 2024 and 31 July 2024.

Sample size

The current study was exploratory in nature and did not involve formal sample size calculations, as there was no specific hypothesis. Our objective was to gather maximum responses within four weeks to ensure a sufficient sample size for assessing awareness and perceptions of AI. We received 404 responses during this period, which was lower than anticipated. Several factors may have contributed to this, such as emails sent through IRIA channels being overlooked or ignored due to inbox overload. Although multiple reminders were sent to minimize non-response bias, it is possible that some radiologists did not find AI relevant to their work or considered the survey topic less interesting or important. The lower response rate indicates that webinars, seminars, or workshops should be used to emphasize the importance of such studies and promote participation.

Ethical considerations

The study was approved by the Institutional Ethics Committee of Sri Aurobindo Institute of Medical Sciences, Indore, with IEC number SAIMS/IEC/54/2024 (Dated 16/01/2024), ensuring adherence to ethical guidelines for human subject research. Owing to the online nature of the survey, consent of the participants was obtained at the beginning of the survey, wherein the participant had to acknowledge the consent statement. Participants' anonymity was protected as no personal information was collected. Only the principal researcher had access to the data, ensuring confidentiality and data security.

Statistical analysis

Descriptive statistics were employed to summarize demographic data and general trends in AI awareness and usage. The chi-square test was utilized to explore associations between demographic factors and attitudes toward AI in radiology. Subgroup analyses were performed to compare perceptions and attitudes between academic and non-academic radiologists and between different age and gender groups. Statistical analysis was performed using R version 4.2.2 (R Core Team (2021). R: A language and environment for statistical computing. R Foundation for Statistical Computing, Vienna, Austria. https://www.R-project.org/). A p-value of <0.05 was considered statistically significant.

## Results

Demographics of respondents

The survey received a total of 404 responses nationwide, with 284 (70.3%) from male participants and 120 (29.7%) from female participants. The age distribution of respondents was diverse, with 118 (29.2%) under 30 years old, 183 (45.3%) aged 30-50, and 103 (25.5%) over 50 years old. Of the total respondents, 241 (59.6%) were affiliated with academic institutions while 163 (40.4%) were non-academic practitioners. Among the 241 respondents from academic settings, there were 31 (12.9%) first-year residents, 32 (13.3%) second-year residents, 41 (17.0%) third-year residents, 31 (12.9%) senior residents, 24 (10.0%) assistant professors, 19 (7.9%) associate/additional professors, and 40 (16.6%) professors. The remainder included fellows, retired, and emeritus doctors. In the non-academic group, 36 (22.4%) were consultants with less than five years of experience, 29 (19%) had 5-10 years of experience, and the majority, 96 (59.6%), had more than 10 years of expertise. Two respondents (0.01%) did not provide information regarding their experience. In terms of practice, 262 (66.4%) of the respondents practiced diagnostic radiology, 3 (0.8%) specialized only in interventional radiology, and 139 (32.8%) were involved in both diagnostic and interventional radiology, as shown in Table 1.

**Table 1:** Demographic characteristics and clinical experience of participants

Characteristics	Number	Percentage (%)
Total Responses	404	100
Gender		
Male	284	70.3
Female	120	29.7
Age		
Under 30 Years Old	118	29.2
30-50 Years Old	183	45.3
Over 50 Years Old	103	25.5
Affiliation		
Academic Institutions	241	59.6
First-Year Residents	31	12.9
Second-Year Residents	32	13.3
Third-Year Residents	41	17
Senior Residents	31	12.9
Assistant Professors	24	10
Associate/Additional Professors	19	7.9
Professors	40	16.6
Non-Academic Practitioners	163	40.4
Working Experience		
<5 Years	36	22.4
5-10 Years	29	19
>10 Years	96	59.6
Type of Practice		
Diagnostic Radiology Practice	262	66.4
Interventional Radiology Only	3	0.8
Both Diagnostic and Interventional Radiology	139	32.8

Prior training, knowledge, and awareness regarding AI

Among the participants, 278 (68.8%) had no prior AI sessions, 111 (27.5%) had attended basic-level sessions, and 15 (3.7%) had attended advanced sessions. Age distribution varied significantly (p=0.005): those under 30 years old were more likely to have no previous AI training (34.53%), while older participants were more prevalent in the advanced session group, with 60% aged 30-50 and 40% over 50 years old. Additionally, doctors from academic institutions were more likely to have attended basic sessions (70.27%) as compared to those with no previous sessions (56.12%) or advanced sessions (46.67%), a statistically significant difference (p=0.021), as shown in Table 2.

**Table 2:** Distribution of participants according to prior training in AI # chi-square test; p-value <0.05 was considered statistically significant

Characteristics	No previous sessions of AI: n=278 (68.8%)	Basic level sessions: n=111 (27.5%)	Advanced level session: n=15 (3.7%)	Chi-square value (degree of freedom)	p-value^#^
Age				14.98 (4)	0.005
Under 30 years old	96 (34.53)	22 (19.82)	0 (0.00)		
30-50 years old	118 (42.45)	56 (50.45)	9 (60.00)		
Over 50 years old	64 (23.02)	33 (29.73)	6 (40.00)		
Affiliation				7.69 (2)	0.021
Academic institutions	156 (56.12)	78 (70.27)	7 (46.67)		
Non-academic institutions	122 (43.89)	33 (29.73)	8 (53.33)		

In our study, 251 respondents (62.1%) were familiar with generative AI, a type of artificial intelligence capable of generating new data similar to the training data it was provided, while 153 respondents (37.9%) were not aware of it.

Attitude

**Figure 1:**
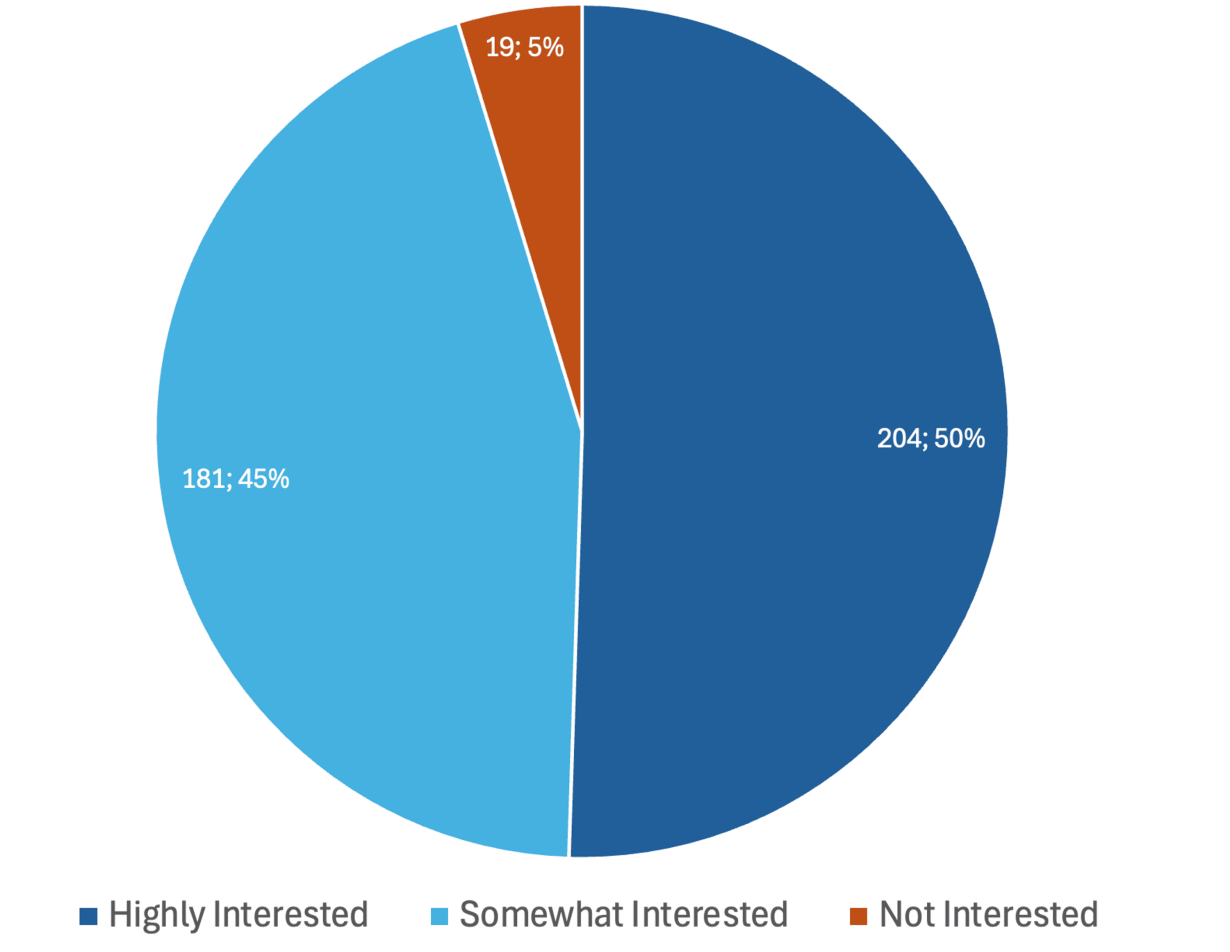
Attitude towards expanding knowledge of AI Question: Are you interested in expanding your knowledge of AI?

Notably, among the 404 respondents, a significant majority, 204 (50.5%), expressed a keen interest in expanding their knowledge of AI, demonstrating a proactive approach and eagerness to stay updated with the latest advancements in their field. Additionally, 181 (44.8%) showed some interest in increasing their understanding of AI, indicating a growing curiosity and openness to learning. Only a small fraction, 19 (4.7%), showed no interest in expanding their knowledge of AI, highlighting the overall positive attitude toward AI adoption in radiology practice. Most respondents (95.3%) identified an educational gap and emphasized the need for more AI-related training and education in medical curricula, particularly among medical residents. Among the 31 first-year residents in our study, 13 were highly interested in AI, 17 were somewhat interested, and 1 was not interested. In contrast, among the 41 third-year residents, 30 were highly interested in AI expertise and 11 were somewhat interested (p = 0.01). This significant difference suggests that third-year residents exhibited more enthusiasm for expanding their knowledge of AI compared to first-year residents. However, there was no significant difference in learning interest between residents and faculty (p=0.16) (Figure 1).

In our study, 350 (86.6%) demonstrated an interest in AI being taught during residency while 54 (13.4%) were not interested in AI teaching during residency.

Perception

**Figure 2:**
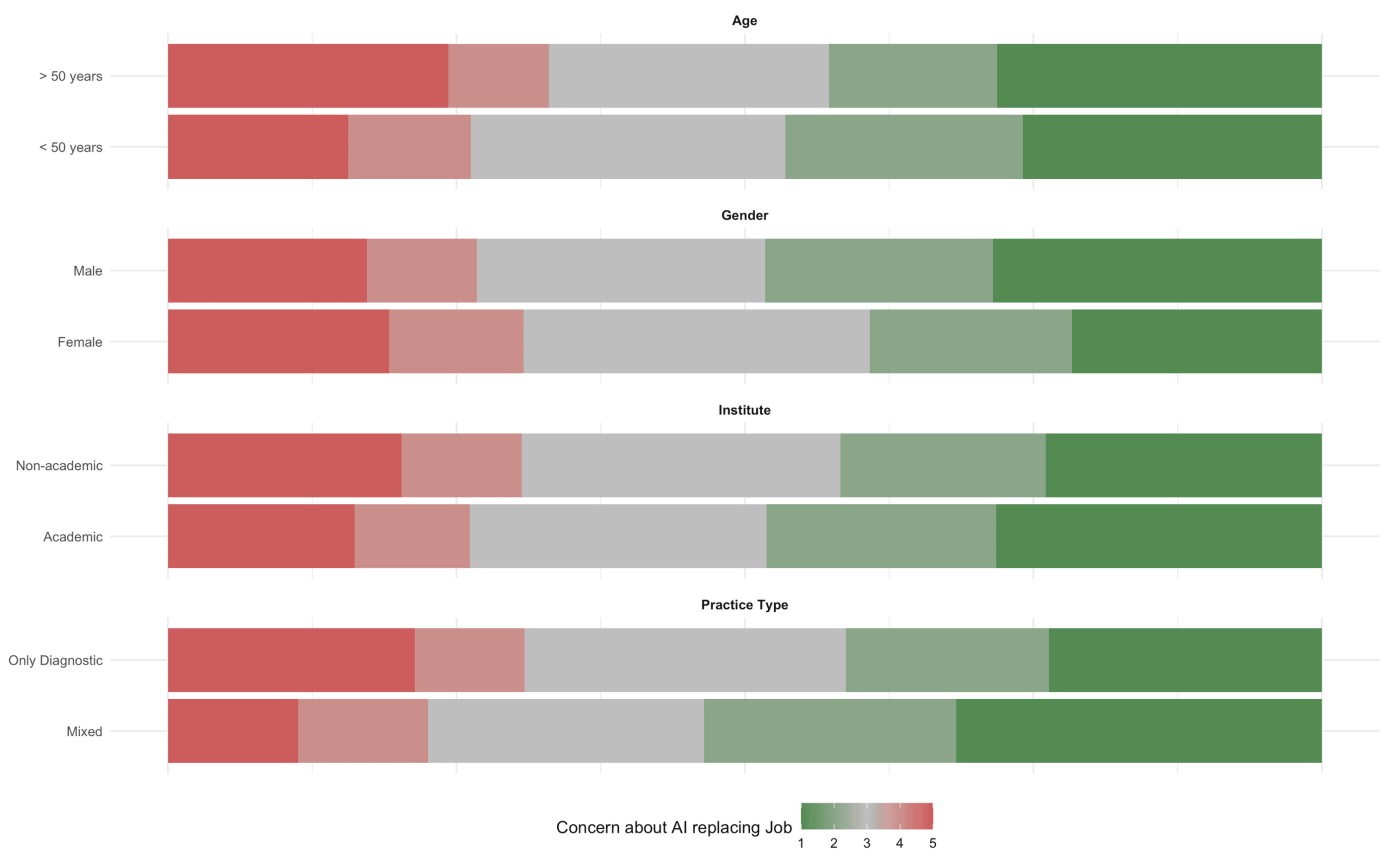
Perception of radiologists regarding losing their jobs to AI

On the Likert scale, 72 respondents (17.8%) were highly concerned, rating their concern as a 5, that AI will take over radiologists' jobs in the next 10 years, indicating a significant level of anxiety. Additionally, 41 respondents (10.1%) rated their concern as a 4, while 107 respondents (26.5%) had a neutral attitude, indicating a mixed level of concern about AI potentially replacing radiologists. Conversely, 77 respondents (19.1%) rated their concern as a 2, and another 107 respondents (26.5%) were confident, rating their concern as a 1, that AI will not replace radiologists in the next 10 years, suggesting a moderate level of confidence that AI will not fully supplant human radiologists. Subgroup analyses show that senior radiologists (age > 50 years) compared to younger, females compared to males, those having academic affiliation compared to non-academic practitioners, and those doing exclusively diagnostic radiology compared to intervention radiology or both are relatively more concerned about AI replacing jobs (Figure 2).

Practice 

Among those who had heard of generative AI, 213 (52.7%) had never used it, 177 (43.8%) had used ChatGPT, and 11 (2.7%) had experience using both ChatGPT and GANs (Generative Adversarial Networks). Additionally, one respondent each reported using co-pilot, PHI-3, and AI-enabled features in sonography machines. In our study, 306 (75.7%) professionals expressed interest in collaborating with software developers to learn and implement AI in their workplace. Meanwhile, 79 (19.6%) were not interested in such collaborations, and 19 (4.7%) were already engaged in AI projects with software developers.

## Discussion

The current study is a one-of-its-kind survey focusing on knowledge, attitudes, perceptions, and practices. While most surveys discuss these aspects among medical students and healthcare professionals from all specialties, this study provides specific data collected from radiologists, a perspective that has been explored in very few studies and different countries. Our findings suggest that most radiologists have a basic awareness of AI and are enthusiastic about incorporating it into their field. Nevertheless, additional training is required to enhance radiologists' utilization of AI in the field. 

Interpretation of results

The survey results indicate a significant awareness, willingness to collaborate with software professionals, and growing adoption of AI in radiology among radiology professionals and residents. The high percentage of respondents acknowledging the importance of AI in radiology underscores the field's rapid evolution. In our research, we discovered that although most radiologists and residents need a comprehensive grasp of the fundamental technical principles behind AI, most are aware of its current discussions in the field of radiology.

A study conducted by Collado-Mesa et al. explored the understanding, attitudes, and implementation of AI among radiology trainees and attending radiologists at a single residency program. With a response rate of 66%, it was found that 29% of the respondents used AI applications, such as computer-assisted detection and voice recognition, in their routine work. However, a notable 36% had not engaged with the scientific literature on AI in the past year. The survey revealed that trainees were more apprehensive about the potential changes AI might bring to their careers and showed a greater willingness to learn about the technology compared to attending radiologists. These findings indicate a significant educational gap, suggesting a need for comprehensive AI-related training to help radiologists effectively integrate these technologies into their practices [8].

A study by the Kenya Association of Radiology indicated that most participants (65.8%) had a fundamental understanding of AI, though fewer had a deep comprehension of more advanced AI-related topics like machine learning and neural networks. The detection application of AI was the most recognized, noted by 37.4% of respondents, while other uses such as segmentation and speech recognition were less commonly acknowledged. Despite this basic understanding, the integration of AI in everyday practice was minimal, with only 12.6% actively using AI tools. Moreover, a significant portion (68.5%) anticipated that AI would substantially alter radiology in the next decade or two, and 67.6% were open to participating in the development and training of ML systems. The study highlights a generally positive attitude toward AI but also points to a need for enhanced educational programs to bridge the gap between awareness and practical application [9].

In a study by Abufadda et al., which included 16 radiologists and 14 radiology residents from six public and private institutions in Jordan, the findings demonstrated a notable awareness and a growing trend toward adopting AI tools among participants [10]. Despite recognizing AI's significance, many respondents lack in-depth technical knowledge of the technology. Similar patterns of awareness but differing levels of technical proficiency and usage were noted in comparative studies from countries like Kenya and Italy. The results highlight the urgent need to incorporate AI training within radiology education programs to improve practical application and maximize AI's clinical advantages. Additionally, the study emphasizes the need to address ethical and data management issues when deploying AI in healthcare settings.

A study by Akinmoladun et al. found that only 12% of the 163 participating radiologists had a good knowledge of AI. However, 58% were willing to implement AI applications in their hospitals, and 60% positively perceived the potential of AI systems in radiology practice [11]. There was a strong correlation between knowledge levels and attitudes, with 82% of those with good knowledge having a positive attitude (P<0.001). Furthermore, 67.6% of participants expressed a willingness to be involved in developing and training machine learning algorithms, reflecting a positive attitude toward AI applications in radiology, despite the low daily utilization of AI applications, which stood at only 12.6%.

An international survey by Huisman et al. involving 1041 respondents from 54 countries revealed that a majority (n = 819, 79%) supported incorporating AI into residency programs [12].

Regarding radiologists' perceptions of AI, Huisman et al. found that 48% of radiologists and residents have an open and proactive attitude toward AI while 38% fear replacement by AI. The study suggested that advanced AI-specific knowledge levels may encourage the adoption of AI in clinical practice, whereas rudimentary knowledge levels seem to inhibit its use. The authors advocated for integrating AI into radiology training curricula to facilitate its clinical adoption [13]. Similarly, a study by Coppola et al. involving 1,032 radiologists from the Italian Society of Medical and Interventional Radiology (SIRM) found that 88.9% were not concerned about job loss due to AI but were more worried about maintaining their professional credibility [14]. Additionally, 73% and 67.9% of the respondents acknowledged the perceived advantages of AI, such as reducing diagnostic errors and optimizing radiologists' workload, respectively. In another study, Chen et al. reported that radiology residents generally held a positive attitude toward AI, with 29.90% agreeing that AI might reduce the demand for radiologists, 72.80% believing that AI improves disease diagnosis, and 78.18% feeling that radiologists should embrace AI ​[7].

AI's impact on radiology practices

Existing research emphasizes AI's ability to augment human capabilities rather than replace them, aligning with the benefits of AI in radiology such as improved diagnostic accuracy and efficiency [15]. However, challenges highlighted by Siala H et al. include the unpredictability of AI decisions and data safeguarding obstacles, underscoring the need for integrating ethics and data governance in AI development and deployment in healthcare [16]. According to the ACR Data Science Institute survey, about 30% of radiologists currently use AI in clinical practice, with its use more prevalent in larger institutions [17]. The primary clinical applications of AI include interpreting cerebral hemorrhages, pulmonary emboli, and mammographic abnormalities. Additionally, 20% of hospitals not currently using AI plan to adopt these technologies within the next 1 to 5 years. A study in the United Arab Emirates observed strong interest among radiologists and radiographers in adopting AI, despite a general lack of expertise, recommending structured training programs through collaboration between professional bodies and educational institutions [18]. The study by Cè M et al. noted an optimistic outlook on AI adoption, especially among young radiologists (<30 years) and seasoned professionals (>60 years), which contrasts with our findings [19]. A 2018 survey by the European Society of Radiology found that only 20% of 675 sampled members were using AI applications; 48% were not using AI, 20% were using AI, and 30% planned to start using it [20]. A follow-up survey in 2022 showed that 40% of 690 members had experience with AI tools in clinical practice, indicating increased awareness and adoption of AI among European radiologists [21].

Limitations of the survey

While our survey provides valuable insights, several limitations must be considered. The sampling strategy adopted was non-random, which may have limited the representativeness of the results, as it is prone to selection bias resulting from higher participation from those who were more interested in or knowledgeable about AI. To address these limitations, future studies should aim to include a larger and more diverse sample to improve the representativeness and generalizability of the results. Furthermore, the self-reported nature of the data may introduce biases, as responses could be influenced by participants' perceptions or recall accuracy. However, not all components of the questionnaire are equally prone to recall bias. A few questions, such as "Have you attended any sessions on AI?", "Have you heard about Generative AI?", or "Have you ever used Generative AI?" may be subject to recall bias, as participants may overestimate or underestimate their experiences.

Wider ramifications

The results of our study emphasize the immediate necessity of integrating AI education into medical training, particularly in the field of radiology. There is a need to develop targeted educational programs encompassing AI technology, ethics, and data management, which aligns with research by Kooten et al. [22]. Among increasing recognition of the importance of AI education for radiologists-in-training, five AI curricula for radiology residents have been implemented [23]. The increasing presence of AI applications in radiology necessitates education to prepare trainees and radiologists as proficient users and stewards of AI technology. The study recommended a course on AI and ML applications in radiology during the residency program, along with continuous medical education (CME) programs for radiologists [9]. Tejani et al. propose content for an AI curriculum, suggest resources for formal and self-directed learning, and discuss the potential of AI-augmented radiology education [24]. This education should encompass technical proficiency, ethical deliberations, and data governance. Collaboration between policymakers and educational institutions is necessary to narrow the divide between the advancements achieved by AI and the existing medical curricula.

Recommendations 

Future research should focus on longitudinal studies to track the evolving perceptions and applications of AI in radiology. In further research, cross-tabulation of findings (e.g., interest in learning AI or concerns about job loss) with prior exposure would yield valuable insights. Additionally, exploring the impact of AI on patient outcomes in radiology can provide more concrete evidence of its benefits and drawbacks. To promote the integration of AI into daily work and maximize its impact on patient care, it is recommended that initiatives be implemented that increase exposure to AI. To harness the full potential of AI in radiology, it is crucial to develop targeted educational programs encompassing AI technology, foster collaborative research that addresses both the technological advancements and the socio-ethical implications of AI in healthcare, and implement policies that promote equitable access to AI resources and training worldwide. The journey of integrating AI into medical science is ongoing, and this integration must be guided by thoughtful consideration of both its possibilities and its challenges.

## Conclusions

The survey results reveal a notable awareness and willingness among radiology professionals and residents to collaborate with software professionals, alongside an increasing adoption of AI in the field. Radiology being one of the first sub-specialties to explore the impact of AI on medical sciences thoroughly, it's essential for radiologists to take on the responsibility of educating their professionals, especially their residents about emerging technologies. This education should focus on a basic understanding of the technical aspects of AI to assess the effectiveness of AI algorithms appropriately and to understand the specific data requirements for various tasks apart from the specific challenges that may arise in the future.

## Appendices

**Table 3:** The study questionnaire

Gender	Male	Female	
Age	<30	30-50	>50
Institute where you are working	Academic	Non-academic	
If you are working in an academic institute	Residents (1st yr, 2nd yr, 3rd yr, senior resident)	Assistant prof, associate prof, prof	Senior consultant, others
If you are working in a non-academic institute	Consultant with < 5 years experience	Consultant with 5-10 years experience	Consultant with >10 years experience
What do you practice	Diagnostic Radiology	Interventional Radiology	Both
Have you attended any sessions on AI	Not at all	Basic level	Advanced level
Have you heard about Generative AI?	Yes	No	
Have you ever used Generative AI?	ChatGPT	GANs	Both/ No/Others
Are you interested in expanding your knowledge on AI	Not interested	Somewhat interested	Highly interested
Do you think AI should be taught during Residency?	Yes	No	
How concerned are you that AI will take over radiologists' jobs in the next 10 years?	1 2 3 4 5		
Do you wish to collaborate with software developer to learn and start AI at your workplace?	Already doing	Interested	Not Interested
